# Tilted grating phase-contrast computed tomography using statistical iterative reconstruction

**DOI:** 10.1038/s41598-018-25075-7

**Published:** 2018-04-26

**Authors:** Lorenz Birnbacher, Manuel Viermetz, Wolfgang Noichl, Sebastian Allner, Andreas Fehringer, Mathias Marschner, Maximilian von Teuffenbach, Marian Willner, Klaus Achterhold, Peter B. Noël, Thomas Koehler, Julia Herzen, Franz Pfeiffer

**Affiliations:** 1 Chair of Biomedical Physics, Department of Physics & Munich School of BioEngineering, Technical University of Munich, 85748 Garching, Germany; 2 MITOS GmbH, 85748 Garching, Germany; 3 Department of Diagnostic and Interventional Radiology, Klinikum rechts der Isar, Technical University of Munich, 81675 Munich, Germany; 4 Philips Research Laboratories, 22335 Hamburg, Germany; 5 Institute of Advanced Study, Technical University of Munich, 85748 Garching, Germany

## Abstract

Grating-based phase-contrast computed tomography (GBPC-CT) enables increased soft tissue differentiation, but often suffers from streak artifacts when performing high-sensitivity GBPC-CT of biomedical samples. Current GBPC-CT setups consist of one-dimensional gratings and hence allow to measure only the differential phase-contrast (DPC) signal perpendicular to the direction of the grating lines. Having access to the full two-dimensional DPC signal can strongly reduce streak artefacts showing up as characteristic horizontal lines in the reconstructed images. GBPC-CT with gratings tilted by 45° around the optical axis, combining opposed projections, and reconstructing with filtered backprojection is one method to retrieve the full three-dimensional DPC signal. This approach improves the quality of the tomographic data as already demonstrated at a synchrotron facility. However, additional processing and interpolation is necessary, and the approach fails when dealing with cone-beam geometry setups. In this work, we employ the tilted grating configuration with a laboratory GBPC-CT setup with cone-beam geometry and use statistical iterative reconstruction (SIR) with a forward model accounting for diagonal grating alignment. Our results show a strong reduction of streak artefacts and significant increase in image quality. In contrast to the prior approach our proposed method can be used in a laboratory environment due to its cone-beam compatibility.

## Introduction

Grating-based phase-contrast computed tomography (GBPC-CT) is an X-ray imaging method utilizing highly sensitive differential phase-contrast (DPC), which offers excellent soft tissue contrast^1–4^. Among different approaches to access the phase signal^5,6^ GBPC-CT has been successfully translated from synchrotron facilities to laboratory X-ray sources with limited coherence^7^.

High-sensitivity GBPT-CT of biomedical samples often suffers from streak artifacts, which are visually comparable to metal artefacts in conventional attenuation imaging and show as characteristic horizontal lines in the phase-contrast data^8^. The origin of the streak artifacts in GBPC-CT lies in a combination of phase-wrapping and the consecutive one-dimensional phase integration of the DPC signal^9^. Additionally, highly absorbing materials like bones cause similar effects due to beam hardening and reduce the data quality of high-sensitivity laboratory GBPC-CT.

Current GBPC-CT setups consist of binary line gratings, which allow only one-dimensional access of the DPC signal. Different methods exist to overcome this downside of grating interferometry exploiting the full two-dimensional phase-gradient signal. One approach is to use two-dimensional gratings instead of line gratings^10^. However, this requires a vastly extended phase-stepping procedure because the stepping has to be performed in two directions or implies reduced spatial resolution when using single-shot methods^11,12^.

Alternatively, one can measure the DPC signal of an object from two directions via rotating the sample around the optical axis by 90° after the first scan, which allows for two-dimensional phase integration^13,14^. This does not only reduce streak artefacts, but also enables the retrieval of orientation sensitive features^15^. Unfortunately, rotating the sample renders tomographic imaging experimentally complicated due to possible misalignment and a second full scan is of limited applicability.

When performing phase-contrast tomography with one-dimensional gratings there is an approach where the gratings are tilted by 45° with respect to the tomographic axis^16^. In detail, a full tomographic scan is executed with the tilted grating configuration over 360° sample rotation. Next, two opposing sample projections, e.g. at rotation angles 0° and 180°, are combined to form DPC projections with gradients in horizontal and vertical direction. Subsequently, the two projections are integrated in two dimensions to retrieve the non-differential phase projections^13^. The combined phase projections are then reconstructed with filtered backprojection using Ram-Lak filters. The results, which were first produced at a synchrotron facility, show an overall improvement of the phase tomogram including reduced streak artefacts^16^.

Trying to reproduce these results revealed two problems: first, the matching and integration of the opposed projections includes additional interpolation steps. The method is prone to fail when dealing with a non-perfectly aligned axis of rotation or weakly absorbing samples. Second, the whole approach cannot be applied to a laboratory setup when dealing with cone beam geometry, since sample structures of two opposing projections are not identical anymore due to the cone perspective.

In this work, we use the tilted grating configuration in combination with statistical iterative reconstruction (SIR) for DPC projections^17^. To achieve this, we retrieve the DPC projections with a laboratory GBPC-CT setup using cone-beam geometry with gratings tilted by 45° by conventional phase retrieval. However, instead of the additional processing outlined above, we employ the SIR algorithm replacing the standard horizontal derivative for upright gratings with a diagonal direction and reconstruct directly the full 360° DPC projections. We compare our results of the proposed method with a non-tilted GBPC-CT configuration using both filtered backprojection and the corresponding SIR algorithm with the horizontal derivative operator. The new method yields a significant quality improvement of the phase-contrast tomogram.

## Results

### Sensitivity direction

**Figure 1:**
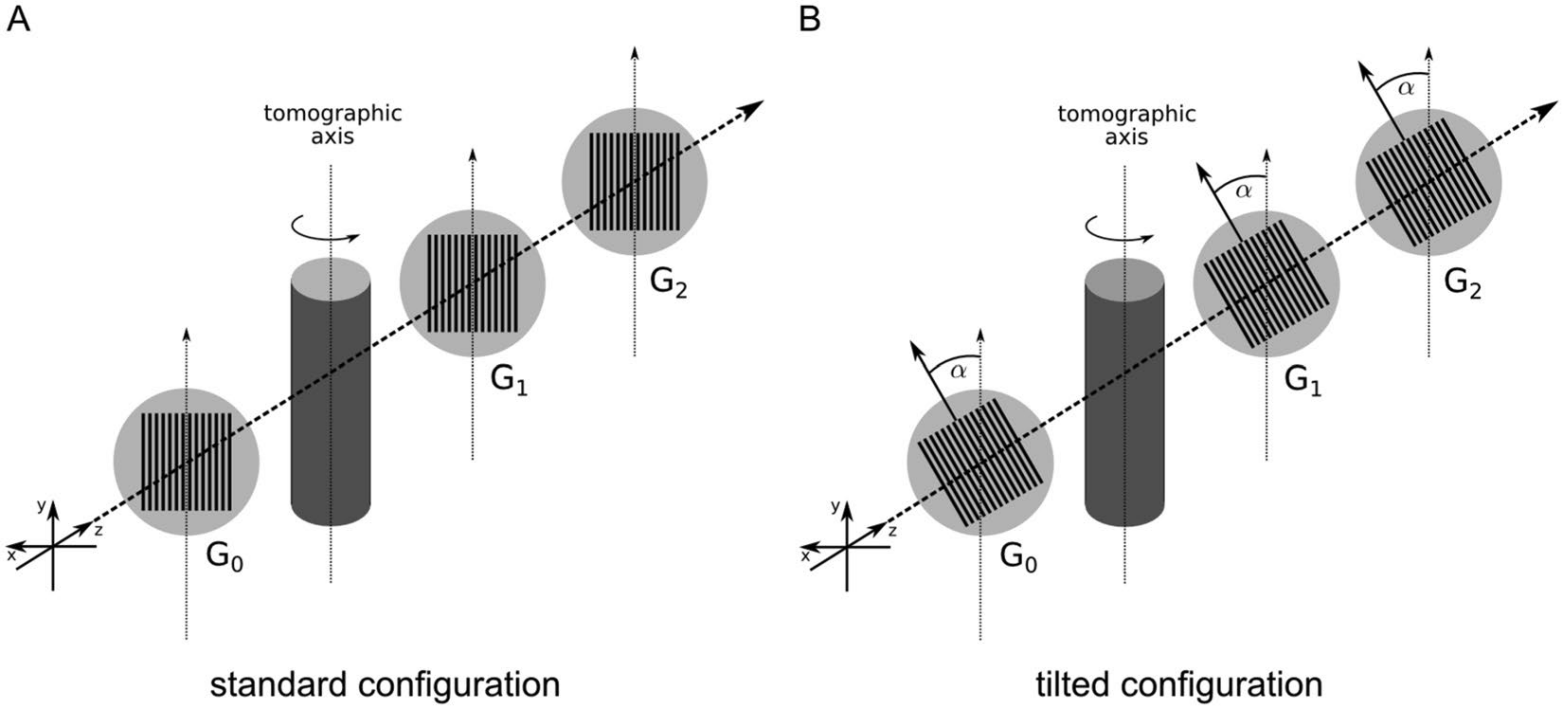
Schematic laboratory GBPC-CT set-up. Subfigure (**A**) shows the standard Talbot-Lau configuration using three gratings with grating lines in parallel to the tomographic axis. Subfigure (**B**) visualizes the tilted grating setup with a rotation of the grating line orientation by *α* = 45° with respect to the optical axis. The sample is indicated by the cylinder centered around the tomographic axis. *G*_0_ is the source grating, *G*_1_ the phase grating, and *G*_2_ the analyzer grating. The X-ray source and the detector are not shown in this figure.

The direction of the sensitivity is perpendicular to the orientation of the grating lines meaning that only components of the refractive index gradient perpendicular to the grating lines can be measured as only these induce a lateral phase-shift of the interference pattern *φ*. The standard GBPC-CT setup configuration is illustrated in Fig. 1(A), in which the orientation of the grating lines is parallel to the tomographic axis. A structure with a component in vertical direction causes the same signal independently of the tomographic angle, whereas a purely horizontal structure would be missed. Figure 1(B) depicts the configuration with a 45°-tilt of the gratings. There, a structure that causes a specific signal at one angular position provides a complementary value at the opposing projection.

**Figure 2:**
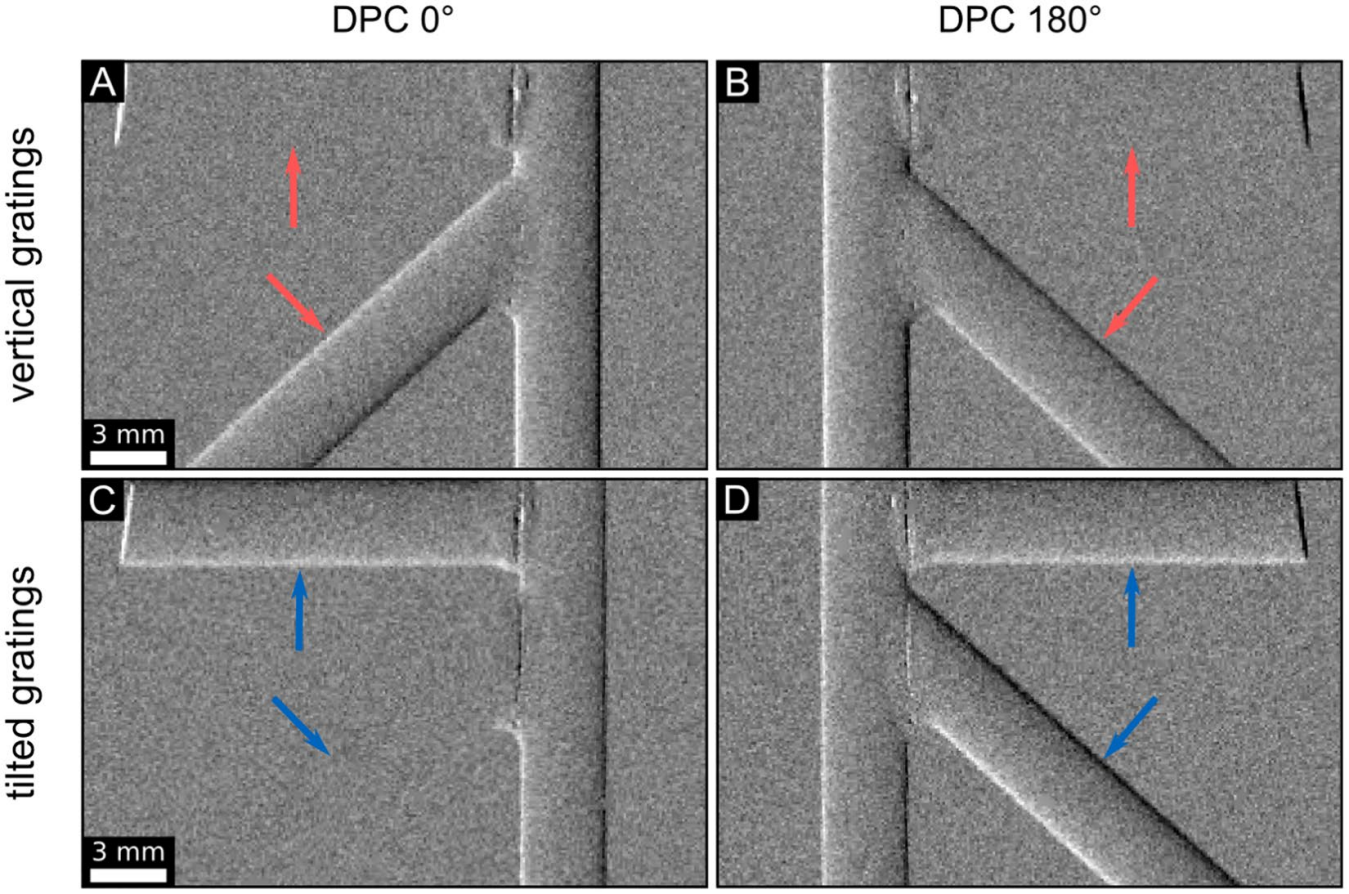
DPC projections of a phantom illustrating the grating sensitivity. The top row was measured with the vertical grating configuration, the bottom row with tilted gratings. Subfigures (**A**) and (**C**) depict the DPC signal of the phantom at 0°. Subfigures (**B**) and (**D**) show the same sample at 180° sample rotation with respect to the tomographic axis. The DPC signal of the full phantom shape is represented by subfigure (**D**). The colored arrows mark typical differences in sensitivity at the same rotational position arising from the different grating orientation. The gray scale of the lateral phase-shift of the interference pattern *φ* is in the linear range of [−0.4, 0.4].

To better visualize this phenomenon we designed a phantom consisting of PMMA (polymethylmethacrylate) rods oriented in vertical, horizontal, and diagonal direction. We measured this phantom at 0° and 180° and retrieved DPC projections (cf. Fig. 2). In Fig. 2(A) and (B) we observe only vertical components of the phantom, which is demonstrated by the vertical and diagonal part of the object. In comparison to that, Fig. 2(C) and (D) emphasize the different situation with the tilted grating configuration. One can obviously see different image content in the projections of the phantom. The vertical and horizontal components of the phantom are retrieved in both projections. However, the diagonal part is missed in Fig. 2(C) and fully observed in Fig. 2(D) because the structure is in parallel to the grating lines in the latter case. Either in the initial or the opposing projection, the information of the refraction signal is obtained, but not totally missed in comparison to the standard configuration.

### Grating-based phase-contrast tomography with tilted gratings

**Figure 3:**
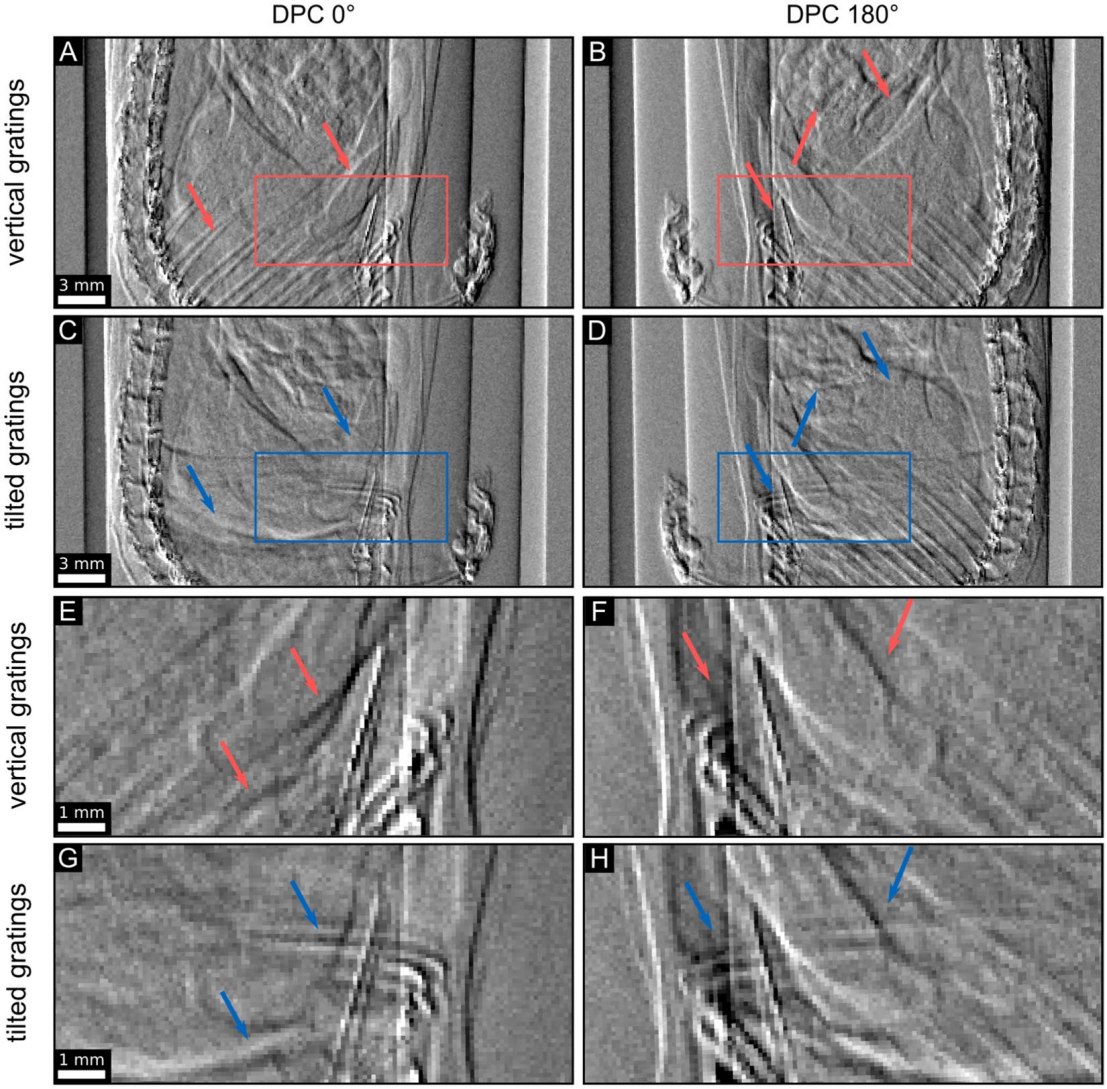
Comparison of non-tilted and tilted DPC projections. Exemplary DPC projections of a mouse sample are shown. The top row represents the measurement with vertical gratings and the second row the tilted grating configuration. Subfigures (**A**) and (**C**) depict the DPC signal of the mouse at rotation angle 0°, subfigures (**B**) and (**D**) represent the same sample at the opposing 180° sample position. The colored arrows mark conspicuous differences at the same rotational position like ribs or intestines. Subfigures (**E**–**H**) represent magnifications of the respective rectangles in subfigures (**A**–**D**). The scale of *φ* is linearly displayed in an interval of [−0.4, 0.4].

We showcase our approach with a GBPC-CT measurement of a mouse sample placed in a plastic container filled with a formalin solution. For comparison, we measured the object twice: first with the standard vertical grating GBPC-CT configuration as visualized in Fig. 1(A) and second with the tilted grating setup (*α* = 45°) and the sample at the exact same positions as illustrated in Fig. 1(B). The resulting DPC projections are depicted by way of example in Fig. 3. Comparing the projections one observes differences in the gained DPC information, indicated with the colored arrows. In Fig. 3(A) the ribs are clearly present, but cannot be found in Fig. 3(C) with the tilted configuration. However, Fig. 3(D) shows information in horizontal direction that would be otherwise lost in the standard configuration (cf. Fig. 3(B)). Magnifications of the areas marked by the rectangles illustrate the differences in further detail (cf. Fig. 3(E–H)).

**Figure 4:**
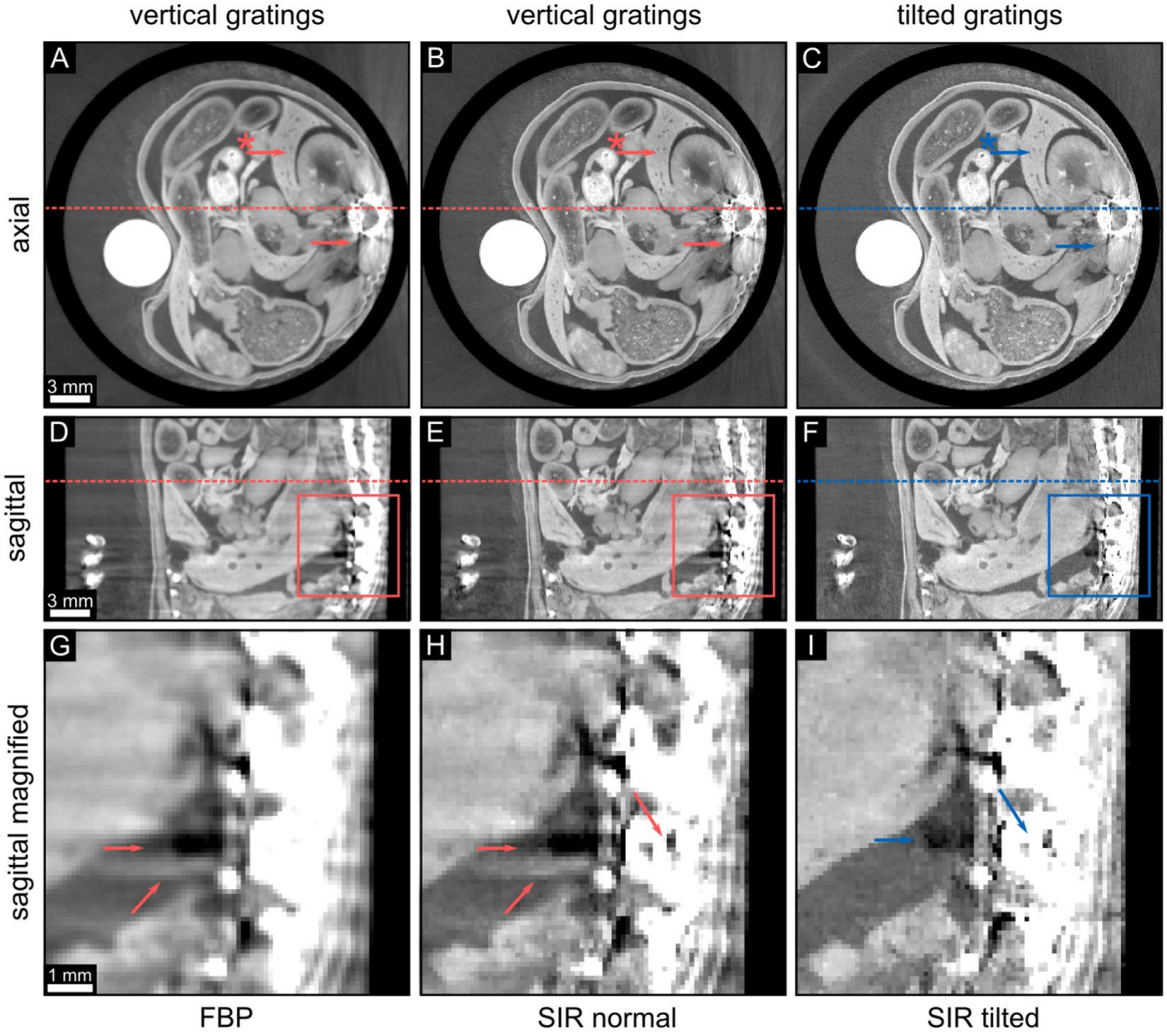
Comparison of non-tilted and tilted phase-contrast tomograms. Different reconstructed slices of the same mouse sample as shown in Fig. 3 are depicted. The left figure part was measured with vertical gratings, the right part was achieved with tilted gratings. The left column shows the FBP reconstruction of the data gained with vertical gratings in axial (**A**) and sagittal slices (**D**) and (**G**). The middle column visualizes the same data set reconstructed with the conventional SIR algorithm. The right column depicts the tilted SIR data set measured with tilted gratings. A magnification of the colored rectangles in the sagittal slices in subfigures (**D**–**F**) is visualized in the bottom row. There, the arrows mark particular differences in the corresponding sagittal slices. The arrows with asterisk mark an area which is highly affected by streaks in the non-tilted configuration (cf. Fig. 4). The dashed lines indicate the corresponding axial or sagittal slice. All phase-contrast values Φ are ranged in a linear scale of [−0.01, 0.03].

The DPC projections with the standard gradient direction were reconstructed with filtered backprojection using the Hilbert filter (cf. left column in Fig. 4) and the SIR algorithm with a horizontal gradient operator (cf. middle column in Fig. 4), which is defined in the methods section. Comparing both data sets the FBP data shows similar horizontal streaks as the SIR data in sagittal view. Note that we use the identical input data for both reconstructions.

The tomographic reconstruction of the measurement with tilted gratings is presented in the right column in Fig. 4. One can observe that there are almost no differences between the data sets in the axial slices (cf. Fig. 4(A–C)) except for less pronounced streaks emerging from the mouse spine. Comparing the corresponding sagittal slices however (cf. Fig. 4(D–F)), the differences become more obvious as the horizontal streaks emerging from the mouse spine are strongly reduced in the tilted data set. Magnifications of the areas framed in Fig. 4(D–F) are displayed in the bottom row of Fig. 4 and visualize the differences more clearly. The arrows mark distinct differences between the sagittal images pronouncing the smooth and almost artefact free area in Fig. 4(I) in contrast to the additional bright or dark features in the non-tilted data (cf. Fig. 4(G) and (H)).

**Figure 5:**
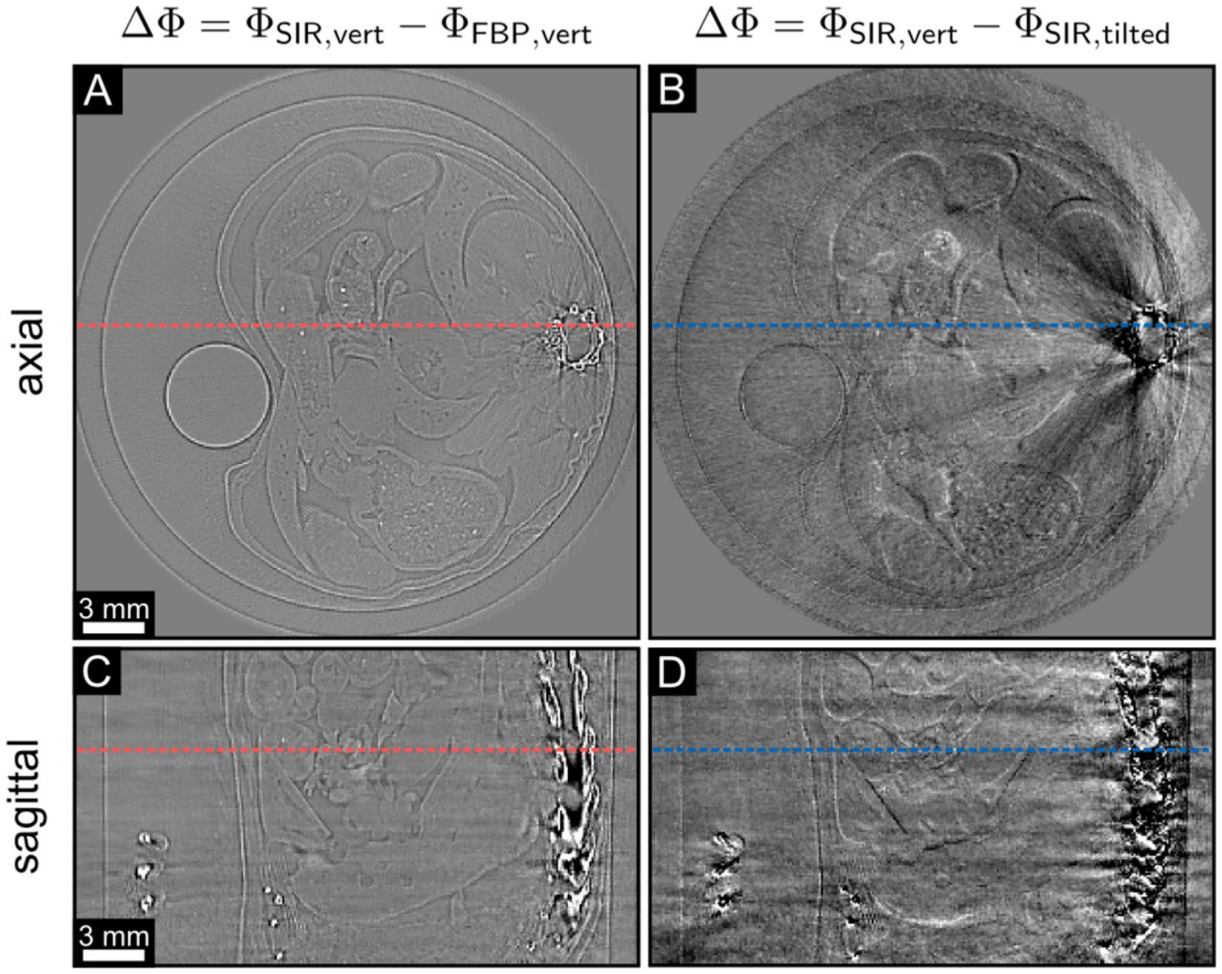
Difference images between different phase-contrast tomograms. The left column shows the differences between the FBP and the SIR algorithm with vertical gratings in axial (**A**) and sagittal view (**C**). The right column shows the differences of the phase-contrast tomograms of the vertical and the tilted grating configuration with SIR, both in axial (**B**) and sagittal view (**D**). The slices are the same as depicted in Fig. 4. The arrows with asterisk represent the same area of the slices shown in Fig. 4. The dashed lines indicate the corresponding axial or sagittal slice. All phase-contrast values Φ are ranged in a linear scale of [−0.01, 0.01].

Figure 5 illustrates difference images between the FBP and SIR data with vertical gratings (cf. Fig. 5(A) and (C)) and between the vertical and tilted SIR slices (cf. Fig. 5(B) and (D)). The shown slices correspond to the respective slices depicted in Fig. 4. In Fig. 5(A) and (C), the difference images between the SIR and the FBP tomograms in vertical configuration allow to assess the effect of SIR in comparison to FBP. The difference images in Fig. 5(B) and (D) visualize strong streaks between SIR with vertical and tilted gratings. The arrow marked with asterisk represents the difference between a quite homogeneous area. Interestingly, no additional significant features could be observed in the slices when comparing tilted with non-tilted data. Solely minor differences at feature borders can be observed (cf. Fig. 5). The reason for this lies in the cone beam perspective. In the case of vertical grating orientation, the cone beam perspective of horizontal features changes for different tomographic angles allowing to detect also vertical components of the horizontal features. In comparison to the tilted-grating configuration, horizontal features are also not missed completely. Additionally, the sample does not provide purely horizontal features. In the case of parallel beam geometry, additional features should be observed when comparing tilted and non-tilted grating configuration^16^.

The Huber regularization strength *λ* was chosen individually to achieve a comparable noise level of the phase-contrast signal Φ at a fixed threshold *γ* of the two SIR reconstructions. The latter was determined by the noise in the PMMA rod–the white circle in Fig. 4(A–C)–in an area of 20 × 20 × 20 voxels with standard deviation values of 6.93 × 10^−4^ for the normal and 6.86 × 10^−4^ for the tilted data. The mean phase-contrast signal Φ in the same volume as before equals 5.13 × 10^−2^ for the FBP, 5.14 × 10^−2^ for the normal SIR, and 5.22 × 10^−2^ for the tilted SIR reconstruction. We therefore conclude, that the GBPC-CT data remains quantitatively reliable by our proposed technique as the slight remaining differences can be explained by reduced streak artefacts and non-identical input data.

## Discussion and Conclusion

In this work, we present a method to reduce streak artifacts occurring in laboratory, polychromatic grating-based phase-contrast computed tomography. High angular sensitivity GBPC-CT is prone to phase-wrapping at large electron density differences, e.g. for bone to water or water to background. Polychromatic GBPC-CT is moreover affected by beam hardening comparable to metal artifacts in conventional CT^8^. Both effects lead to streak artefacts. The tilted grating approach in combination with statistical iterative reconstruction improves the quality of GBPC-CT as the extent of streak artefacts is enormously reduced and thus enables high-sensitivity GBPC-CT of biomedical samples containing highly absorbing material like e.g. bones.

Experimentally, the technique is simple to realise as solely the gratings have to be tilted around the optical axis. The parameters of the experiment are the same compared to standard phase-contrast tomography, especially the duration of the tomographic scan is unchanged and the processing of the data does not differ. The diagonal derivative operator in the iterative reconstruction framework is implemented by exchanging the horizontal derivative operator.

Other methods for streak artefact reduction working for DPC imaging use phase-unwrapping with energy resolving detectors^18,19^ or an iterative bone-artefact removal algorithm^17^, but are more complicated than the presented approach. In contrast to the tilted gratings method presented at a synchrotron facility^16^, no additional processing is necessary with the approach illustrated in this work. Potential errors due to sample alignment mismatch and additional interpolation in the processing can be avoided. Moreover, our method works in cone beam geometry and increases the range of possible high-sensitivity GBPC-CT applications in a laboratory environment. However, iterative reconstruction must be used in the laboratory tilted grating configuration and usually takes longer than filtered backprojection.

Additionally, an implementation in advanced reconstruction methods like intensity-based SIR is possible^20^. Furthermore, the modified SIR algorithm can be employed at other one-dimensional phase-contrast imaging techniques with differential character like analyzer based or diffraction enhanced imaging^6^. The tilted grating approach can also be beneficial in anisotropic x-ray dark-field tomography^21^.

## Methods

### Experimental setup

The laboratory GBPC-CT setup (or Talbot-Lau interferometer) consists of an X-ray source, three gratings, and an X-ray detector described in full detail in ref.^22^. The X-ray source is a rotating anode of type Enraf Nonius FR591 with a molybdenum target operating at 40 kVp and 70 mA. We use a Dectris Pilatus II 100K single-photon counting detector with a 1 mm thick silicon sensor and 487 × 195 pixels of 172 × 172 μm^2^ pixel size. The setup is designed for a phase shift of *π* with a design energy of 27 keV and for inter-grating distances of 85.7 cm. The cone angle is approximately 11 mrad. The gratings are binary line gratings with a period of 5.4 μm on silicon wafers. The first grating, the so-called source grating, is an absorption grating providing sufficient transverse coherence. The second grating, the phase-grating, creates a periodic phase modulation. The last grating, the analyzer grating, enables resolving the interference pattern whose period is usually much smaller than the detector pixel pitch. All gratings were developed and fabricated by the Institut für Mikrostrukturtechnik, Karlsruhe Institute of Technology (Karlsruhe, Germany). In order to retrieve the DPC signal besides the conventional attenuation and the dark-field signal–which are not considered here–we use the phase-stepping approach for both the standard and tilted configuration of the gratings^4^. The scan parameters were identical in both cases, namely 11 phase-steps, 5 s exposure time per phase step. Also, the same processing including 2D polynomial ramp removal^23^ and flatfield correction was used. In order to reduce the effect of phase-wrapping, all samples were immersed in a water container^24^. For the tomographic scans, 1200 equidistant projection angles were taken.

### Tomographic reconstruction

After DPC data acquisition, the established way of reconstructing the projections in standard grating configuration is the use of filtered backprojection (FBP) with a Hilbert filter to account for the differential nature of the phase-contrast signal^25^.

The SIR algorithm used for our novel tilted approach and the standard configuration is based on the maximum a-posteriori principle, which considers statistical properties of the projection data and enforces prior knowledge about the object^17,26–29^.

Several steps are necessary for advanced iterative reconstruction. First, the data has to be discretized similarly to FBP reconstruction. Next, prior knowledge and a combination of forward and data model have to be included. The forward model generates a physical simulation of the expected experiment and the data model calculates the log-likelihood minimization of the difference between measurement data and the forward projection of the calculated data model. More information about iterative reconstruction can be found in refs^26,30^.

In further detail, the idea is to determine the tomographic volume **x** of an object after measuring experimentally a set of projections **p**. Thereby, we minimize the cost function

1 $${\rm{L}}={\Vert \begin{array}{c}\nabla {\rm{A}}{\bf{x}}-{\bf{p}}\end{array}\Vert }_{{\bf{w}}}^{2}+\lambda {{\rm{R}}}_{{\rm{Huber}}}({\bf{x}},\gamma ),$$

with **p** denoting the retrieved DPC projection data and A being the forward projection system matrix. The system matrix ∇A includes the setup cone-beam geometry. The statistical weight of the projection data **w** is determined by the least square processing of the raw projections^17^ and *λ* R_Huber_ (**x**, *γ*) represents the Huber regularization with parameters *λ* and *γ* constraining the otherwise ill-posed reconstruction problem to a smooth solution. The derivative operator ∇ for the standard configuration can be described by a convolution with the forward difference kernel, which is

2 $${\nabla }_{{\rm{horiz}}}=[\begin{array}{cc}-1 & +1\end{array}]$$

in the case of a horizontal gradient. For the two possible tilted grating configurations with an angle of *α* = ±45° the derivative operator ∇ equals

3 $${\nabla }_{{\rm{diag}}}=\frac{1}{\sqrt{2}}[\begin{array}{cc}0 & +1\\ -1 & 0\end{array}]\,{\rm{and}}\,\frac{1}{\sqrt{2}}[\begin{array}{cc}+1 & 0\\ 0 & -1\end{array}],$$

which is also known as Roberts cross operator. In principle other angles are possible, but of limited use as *α* = 45° is the optimum choice for this approach due to its signal complementarity. For the minimization, we employ a limited-memory Broyden-Fletcher-Goldfarb-Shanno (L-BFGS) algorithm^31,32^ until the difference of 20 consecutive iterations is less than 1 × 10^−3 ^^33^. The Huber regularization parameters were chosen empirically. To render SIR reconstructions comparable, the threshold parameter *γ* was fixed (*γ* = 10^−3^) to achieve a comparable edge-sharpness, whereas the regularization strength *λ* was adapted individually (*λ*_vertical_ = 1.5 × 10^−1^ and *λ*_tilted_ = 3.0 × 10^−3^) to reach an overall comparable noise level in the image.

The Huber regularization R_Huber_ (**x**, *γ*) is a potential function, which penalizes different neighbouring pixel values. The Huber function can be defined as

4 $${{\rm{R}}}_{{\rm{Huber}}}({\bf{x}},\gamma )=\sum _{i}\sum _{j\in {N}_{i}}{m}_{ij}\{\begin{array}{ll}\frac{{({x}_{i}-{x}_{j})}^{2}}{2{\gamma }^{2}} & {\rm{for}}\,\,|{x}_{i}-{x}_{j}|\,\le \gamma \\ \frac{|{x}_{i}-{x}_{j}|-\gamma \mathrm{/2}}{\gamma } & {\rm{for}}\,|{x}_{i}-{x}_{j}|\, > \gamma ,\end{array}$$

with the regularization threshold *γ*, the included neighbouring pixels *N*_*i*_ with respect to pixel *i*, and the weights *m*_*ij*_ depending on the distance of the voxels^34,35^. Depending on *γ*, small difference pixel values are weighted quadratically (|*f*_*i*_ − *f*_*j*_| ≤ *γ*) and more differing pixel values are weighted linearly, which preserves edge discontinuity (|*f*_*i*_ − *f*_*j*_| > *γ*).

After reconstruction, the gained phase-contrast signal Φ can be related to the refractive index decrement *δ*, which is in turn proportional to the electron density^36,37^.

### Data availability statement

The experimental data is available from the corresponding author (LB) on request.
